# Editorial

**Published:** 1864-03

**Authors:** 


### Editorial.

ILLINOIS STATE MEDICAL SOCIETY.—The next regular Annual Meeting of this Society will be held in this city (Chicago) on the first Tuesday in May next, commencing at 10 o'clock A.M. The Committee of Arrangements are making all necessary local preparations for the accommodation of the Society. We can assure the Profession, in every part of the State, that all who may come to the meeting will be cordially received. Each local society is entitled to one delegate for every ten members, and an additional delegate for a fraction of more than half of ten. Every regularly organized medical college is entitled to two delegates; and each hospital for the treatment of the sick, to one delegate. Besides delegates, the constitution provides for the election at each Annual Meeting of permanent members, and of members by invitation.

Hence, we hope all who feel an interest in the advancement of the profession will attend the coming meeting. In addition to reports and papers from Standing and Special Committees, on a variety of important subjects, there are several questions of great interest that might be properly and profitably considered by the Society. The practicability and desirableness of establishing a single Board of Examiners in each State, for the examination of all candidates for admission into the profession, is a proposition again undergoing discussion in the New York State Medical Society and elsewhere. And it is quite probable that it will be brought prominently before the next meeting of the American Medical Association, where the delegates from our own State Society may be called upon to record their votes concerning it. Is the present mode of recommending the use of alcoholic beverages in medical practice, as guarded and free from injurious consequences as it should be? This is another question of great practical importance, and worthy of more serious consideration than it has yet received.

We anticipate a full and interesting meeting of the Society.

---

CHICAGO MEDICAL COLLEGE—MEDICAL DEPARTMENT LIND UNIVERSITY.—The Fifth Annual Commencement of this institution was held in the Hall of the College, on the 1st instant. The Hall was well filled with an attentive and appreciative audience. The degree of Doctor of Medicine was conferred on seventeen candidates, who, with their diplomas, received from the President of the Faculty, Prof. H. A. JOHNSON, a short but eloquent and impressive charge concerning the privileges and responsibilities of their new position. Prof. H. WING then delivered a highly interesting and instructive Valedictory Address, which will be published in full in the next number of the EXAMINER. At the close of the address, it was announced that the regular summer course of instruction would commence on the second Monday in March, and continue until the first Monday in July. The audience was then dismissed, and the students, graduates, and Faculty, with the invited guests, repaired to the residence of Prof. H. A. JOHNSON, where they found an elegant entertainment, and enjoyed a most pleasing social interview until a late hour of the night. The college term, thus pleasantly closed, had been a highly prosperous and satisfactory one. The whole number of matriculants was eighty-nine, and the number of graduates seventeen.

The attendance in all the departments, Junior, Senior, and Clinical, had been uniform and punctual to the last day of the session. The possession of good and permanent college building and grounds; the long period of Annual Lectures; the addition of the Summer Recitation and Clinical Course; the proper arrangement of courses in a progressive instead of a repetitional order; and the complete identification of hospital clinical instruction as a necessary part of both the winter and summer terms, makes this institution the most complete and comprehensive medical school in this country.

---

CHICAGO, March 1, 1864.

DR. DAVIS, EDITOR EXAMINER:—*Dear Sir:*—Dr. A. M. JOHNSON, of Peoria, recently stated to me a number of facts going to show that the bark of the common sugar maple is highly efficacious in the treatment of intermittents. I state none of the facts, because it is the object of this communication merely to throw out the suggestion, to be tested by those who have opportunity and inclination.

I am not unaware of the many sources of fallacy in a point like this, but I have great respect for Dr. JOHNSON'S opinion, and he was very sanguine that the most decided results would be obtained by a trial of the article.

The directions for use are the old popular formula:—Take a pugillum of the bark, steep it in a pint of water, and drink freely of the infusion.

Yours, &c., H. WING.

---

AMERICAN MEDICAL ASSOCIATION.—The Fifteenth Annual Meeting of the "American Medical Association" will be held in the city of New York, commencing Tuesday, June 7th, 1864, at 10 o'clock A.M. Proprietors of medical journals throughout the United States and their Territories are respectfully requested to insert the above notice in their issues.

H. A. JOHNSON,<br>GUIDO FURMAN, } *Secretarieo.*

We take pleasure in calling attention to the above notice of the next Annual Meeting of the American Medical Association.

The Meeting will, undoubtedly, be one of the largest and most important that has been held since the formation of the Association. We think the committees, appointed at the last meeting, will, in most instances, be prepared to report; and consequently there will be an unusual number of valuable papers for consideration. It is highly probable, also, that some of the important questions connected with medical education, will be presented in a manner to command more definite and practical action than heretofore. Let the profession of the North-west see to it, that they are well represented.

Every city, county, district, and State Society is entitled to one delegate for every ten of its members; each medical college, and hospital containing one hundred beds for the sick, to two delegates; and all hospitals with less than one hundred beds, to one delegate. We earnestly urge all the societies, colleges, and hospitals, whether civil or military, to select such delegates as will attend the meeting, and send as early as possible a list of their names, either to the local secretary, GUIDO FURMAN, M.D., New York, or to the chairman of the committee of arrangements.

---

### LETTER FROM DR. W. N. COTE.

PARIS, Feb. 4, 1864.

### CHEAP FOOD IN PARIS.

The Philanthropic Society of Paris has published its report for 1862. From the ten cheap food kitchens established in different parts of the capital, it distributed 290,016 portions of food, the produce of which was 11,709 francs, and the expenses 22,522 francs. In the six dispensaries, 1,775 patients received medical assistance, whilst 1,659 gratuitous consultations were given. Out of that number of sick, 81 died, being a smaller proportion than takes place in the hospitals. The expenses of those dispensaries for 1862, amounted to 41,985 francs, including medicines and bath, or an average of about 24 francs per patient. The budget of the Society for 1862, is stated as follows:—The balance in hand was 35,508 francs, and the receipts 66,665 francs, making a total of 102,173 francs. The general expenses amounted to 77,856 francs, showing a surplus of receipts of 24,317 francs. As you are no doubt aware, five francs equal a dollar.

### TREATMENT OF BRONCHOCELE.

Bronchocele, or goitre—a disease which is marked by a tumor on the forepart of the neck, seated between the trachea and skin, and usually occupying the thyroid gland—is very often met with, and, on account of the inconvenience it generally causes, has been a subject of much attention on the part of surgeons. Various methods of treatment have been instituted against this common disorder. Some surgeons have resorted to extirpation, an operation first recommended by Celcus, and which has sometimes proved successful. But it is quite unsafe to attempt its removal with the knife, on account of the enlarged state of its arteries and its vicinity. Palfin, in his Anatomie Chirurgicale; Dr. Bell, in his Surgical Works; Desault, Bonetus, Severinus, and Dupuytren, testify to the danger of this operation, and many patients have died in the hands of the most expert and enterprising surgeons. Cases are recorded, however, where operations of the kind have been attended with success, but it must be confessed, they are extremely rare, and extirpation should be resorted to only when bronchocele has become so large as to endanger suffocation.

Dr. Blizzard, of London, recommends the tying up of the great arteries in the vicinity of the tumor, from which it chiefly derives support. This operation is preferable to excision of the thyroid gland, inasmuch as it presents comparatively but little danger. Internally, a remedy which has proved very efficacious in this disease is burnt sponge, administered usually in the form of lozenges, which consist of cinnamon, gum arabic, syrup, and calcined sponge. It may be given in the form of powder, mixed with honey and other materials, or the simple decoction of the sponge, as recommended by Herrenschward, of Berne, in Switzerland. Many persons laboring under bronchocele, have been cured by this remedy, some of whom began to suffer much, and to be seriously alarmed on account of the difficulty of respiration and deglutition with which their complaint was attended. Sulphate of potash, continued for several weeks in large doses, is said to have effected many cures, in the hands of Fodere and other practitioners. It should be given dissolved in water, in the proportion of thirty grains to a quart daily. Besides burnt sponge and sulphate of potash, mercury, pumice stone, muriate of barytes, egg-shells, muriate of lime, digitalis, muriate of iron, belladonna, electricity, pressure, frictions, issues, setons, blisters, and caustic, have been employed against bronchocele. With what success does not appear. I now see by the *Journal de Societie de Medicine de Rouen*, that *iodine* is highly recommended as a remedy for goitre. It is employed according to the following formula:—

Take pure glycerine, 1000 grammes=2 lbs.
Common dry soap (pulverized) 50 grammes=1½ oz.
Dry powder of Iodate of Potassium, 130 grammes= 4 oz.
Essence of sweet almonds, 2 grammes=40 gr.

To be rubbed over the tumor as often as necessary. At the same time the patient should take internally, solution of the tincture of iodine, made of four grammes of iodate of potassium, dissolved in thirty of the tincture. Three, four, or five drops may be given at every meal. At night, before going to bed, a pinch of the powder of calcined sponge may be administered, good diet, dry habitation, exposure to the sun, sea baths. Such is the treatment usually followed in the Department of the Seine Inféríure. Iodine was first introduced into practice by Dr. Coindet, of Geneva, who employed it with success as a remedy for bronchocele.

ACADEMY OF SCIENCES—PRIZES.

The Academy of Sciences lately held its annual session at the Institute, Dr. Velpea presiding. The prize for *Experimental Physiology* was given to Dr. Moreno. For *Medicine and Surgery*, the Academy awarded to Dr. Chassaignac a prize of 2,500 francs, and to Drs. Bourdon, Cahen, Debont, and Gallois, an honorable mention, with 1,500 francs for each. For *Insalubrious Arts* a prize of 2,500 francs was given to M. Grimaux de Caux, for his work "On the Supply of Water to Towns and Rural Habitations." A prize of 2,500 francs to M. Guignet, for the preparation of non-injurious green for printing on tissues; and a recompense of 1,500 francs to M. Bouffe, for having discovered a substitute for arsenical green in the manufacture of artificial flowers.

The following are the prizes on medical subjects proposed for this year and the ensuing ones by the Academy, the papers in all cases to be sent in headed with some motto, to be repeated on a sealed envelope containing the name of the author. 5,000 fr. "To give a complete History of Pellagra," (March 31st, 1864.) 5,000 fr. "On the Application of Electricity to Therapeutics, (March 31st, 1865). 20,000 fr., the Academy and Emperor's prize, "For Method of Preserving Members by Preserving the Periostem," (March 31st, 1866). Lastly, the Breant prize of 100,000 fr. for the discovery of an unquestionable specific against cholera, or, in default of this, a prize of 4,000 fr.

to the competitor who can prove that there exist in the air substances which may materially contribute to the propagation of epidemical diseases.

### SENILE CATARACT.

At the Medico-Surgical Congress of Rouen, Dr. Wecker read a paper on *The Surest Method of Removing Senile Cataract.* You are aware that there are two or three different operations now in use—*couching* or *depression* of the cataract, an operation practised, it is thought, long before the time of Celcus, and which consists in lacerating freely, but cautiously, with the point of a needle, the anterior capsule of the lens, and then pressing the lens itself downward and backward, and lodging it in the vitreous humor. Another method is that denominated by Sir William Adams, as the *absorbent practice*, and said to have originated with Mr. Pott. The aqueous humor, having been found to have a solvent power, advantage is taken of this circumstance, by pushing fragments of the lens which happen to be detached during the operation of couching into the anterior chamber. Two operations are in use, each founded on the absorbent principle—the *anterior* and *posterior*—the former being performed by penetrating the cornea at its lower and outer part about a line anterior to its union with the sclerotic coat, whilst in the posterior operation the opening is made in the schlerotic coat instead of the cornea. There remains another operation which is often resorted to—*extraction* of the cataract, which is performed by a knife instead of a needle. The knife is introduced above the equator of the cornea, and about a quarter of a line anterior to its junction with the sclerotica, with the edge downward, passing it slowly and steadily along through the anterior chamber until its point emerges at the inner edge of the cornea. The section of the cornea being made, the aqueous humor is discharged, and the anterior capsule of the lens is then cautiously lacerated precisely in its centre, and brought out through the opening made in the cornea. As soon as the lens is removed, the flap of the cornea is adjusted, the lids closed, and a bandage applied lightly over both eyes. Hitherto surgeons have been exceedingly cautious against wounding the iris, and in order to guard against this, Baron Wenzel has recommended friction of the cornea with the end of the finger during the passage of the knife, an expedient which causes the iris to retire immediately from the edge of the knife, and remain so long as the friction is continued.

Dr. Wecker recommends the following method of extraction, which consists in making the section of the cornea near the conjunctival angle so as to include the most vascular portions of that membrane, and their excising with curved scissors from three to four millimetres of the iris. This is what Dr. Wecker calls modified method of extraction. Iridectomy may be practiced some time before extraction, so that the wound of the iris be wholly cicatrized when the moment comes for operating on the cataract. W. N. COTE.

—*Medical and Surgical Reporter.*

### FORMULA FOR A SOLUTION OF BROMINE.

By Dr. J. Lawrence Smith, Professor of Chemistry in the Medical Department of the University of Louisville.

The frequent demand for bromine from the Louisville Chemical Works, which are under my direction, induced me to inquire for what purpose it was used, and I learned that it was being employed as a therapeutic agent, especially in the form of vapor mixed with air as a purifier of the atmosphere of hospitals, where erysipelas, gangrene, small-pox, &c., existed, and also internally in certain affections of the throat. Knowing full well the inconvenience of the use of the substance in the form called for, I at once undertook to compound a solution which would meet the ends required, and be more convenient for any therapeutical use to which uncombined bromine might be applied. From the slight solubility of bromine, any attempts to dissolve it in water would give too dilute and bulky a solution, the natural suggestion, therefore, was to use but little water and facilitate its solubility by adding bromide of potassium; at first the following proportions were used:—1 troy ounce of bromine, 120 grains bromide of potassium, and 1 fluid-ounce of distilled water; the formula left a small quantity of bromine undissolved, and the solution was too concentrated. After varying the proportions in different ways, I have settled on the following as the most convenient formula:—

℞.—Bromine, 1 troy ounce; bromide of potassium, 160 grains; distilled water, q. s. to make four fluid-ounces of the whole mixture.

Dissolve the bromide of potassium in about two fluid-ounces of water in an eight ounce bottle, then add the bromine, agitate gently until the solution is complte, then add water enough to bring the whole to four fluid-ounces.

This mixture forms a very dark red solution, evolving strong fumes of bromine, and readily soluble in any additional quantity of water.

I have given this formula as one that will doubtless recommend itself to those of the medical profession engaged in using bromine, and it is already being used by the medical profession of this place.—*American Journal of Medical Science, April,* 1863, *p.* 385.

### ON THE USE OF COLLODION WITH GLYCERINE IN ERYSIPELAS.

The substitution of a glycerinated collodion for the common collodion will completely prevent the cracking of the skin and other unpleasant consequences which often occur when the latter is used as a dressing in erysipelas of the face.

The manner in which it should be prepared, according to Dr. W. Abbotts Smith's recent small work "On Glycerine, and its Uses in Medicine, Surgery, and Pharmacy," p. 30, is by adding two parts of glycerine to one hundred parts of common collodion. The addition of this small proportion of glycerine is sufficient to impart considerable suppleness to the collodion, and to prevent its dragging upon and cracking the delicate tissues to which it is applied.

[Equal parts of collodion and castor oil are recommended by another writer.]—*Lancet, October* 31, 1863, *p.* 527.

Professional Dignity in France.—Our readers have not forgotten how certain parties have of late been summarily dealt with by the Medical Council and the College of Surgeons for unworthy conduct. A measure of the same kind has lately been adopted by the Academy of Medicine of Paris respecting a medical man named Priou, practicing at Nantes. This individual was a Corresponent of the Academy, and had lately, at the Medical Congress of Rouen, bills posted all over the town with advertisements of the most reprehensible kind. The members of the Congress protested very energetically, and the Academy has at last taken up the matter. It has been decided that Priou, for this forgetfulness of professional dignity, should be erased from the list of correspondents.

Perseverence.—The chair of Surgery of the Faculty of Turin was lately competed for by Messrs. Pacchiotti and Bottini. The former beat his adversary and obtained the professorship, for which he had competed *five times* previously.

